# Long-Term Change in Vitamin D Status and its Association With Change in Total Hip Bone Mineral Density in Older Women: A Population-Based Cohort Study

**DOI:** 10.1016/j.mayocpiqo.2025.100681

**Published:** 2025-12-03

**Authors:** Karl Michaëlsson, Håkan Melhus, Liisa Byberg, Eva Warensjö Lemming, Bodil Svennblad, Jonas Höijer, Hannah L. Brooke

**Affiliations:** a Medical Epidemiology, Department of Surgical Sciences, Uppsala University, Uppsala Sweden; b Clinical Pharmacology, Department of Medical Sciences, Uppsala University, Uppsala Sweden

## Abstract

**Objective:**

To examine if long-term constant low vitamin D status in the sunny season has a greater impact on bone mineral density (BMD) over time than long-term constant low vitamin D status in the dark season.

**Patients and Methods:**

In a longitudinal cohort study conducted from November 3, 2003 to May 22, 2019, 1802 Swedish women living in Uppsala County (latitude 58^o^N) (mean baseline age of 65 years and average follow-up of 12 years) had vitamin D status measured by serum 25-hydroxyvitamin D concentration (S-25OHD). Participants were stratified by season of blood draw (dark [November-April] vs sunny [May-October]). We examined the association of long-term stable season-specific S-25OHD with 12-year changes in total hip BMD, measured by dual-energy x-ray absorptiometry, and investigated if increasing S-25OHD during follow-up influenced changes in BMD by baseline S-25OHD levels and season.

**Results:**

Compared with longitudinally sunny season constant S-25OHD>70 nmol/L, women with sunny season constant S-25OHD<40 nmol/L displayed 10.0% (95% CI,3.8%-16.1%) lower total hip BMD at follow-up. No difference in BMD was observed by dark season S-25OHD. Among women with baseline sunny season S-25OHD<45 nmol/L, each 20 nmol/L increase in S-25OHD during follow-up was associated with a 2.5% increase in hip BMD (95% CI,0.5-4.6). This estimate was attenuated when increasing the low S-25OHD cut-off and was not observed with dark season samples.

**Conclusion:**

Women with sunny season S-25OHD<40-50 nmol/L are a likely target group for vitamin D interventions to improve BMD. Blood samples taken during the dark season are less informative for determining future bone health.

## Graphical abstract

**Figure:**
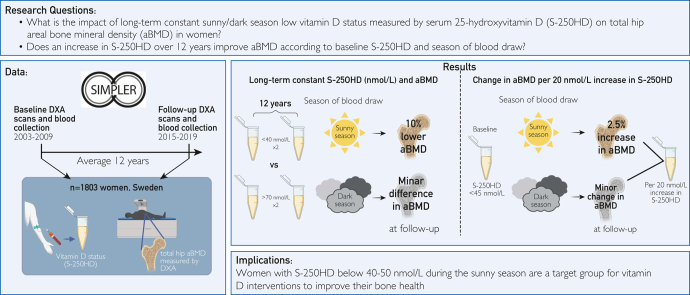

One of the long-recommended cornerstones for preventing fragility fractures is maintaining a normal vitamin D status, although the precise level for categorizing vitamin D insufficiency has been debated over the past few decades. Vitamin D promotes increased intestinal calcium absorption and renal calcium conservation to sustain adequate levels of ionized calcium in the serum. During periods of vitamin D deficiency, bone resorption increases due to decreased active calcium absorption, leading to a reduction in bone mineral density (BMD) and a higher risk of osteoporosis with increased rates of fragility fractures.^1^

Effective prevention of fragility fractures is essential. It is estimated that approximately 180 million men and women worldwide will experience a fragility fracture each year, with older adults accounting for the majority of these fractures. Fragility fractures have risen by 70% in the past two decades.^1^ These fractures significantly affect quality of life; only one-third of patients regain their pre-fracture level of function.^2^

Approximately half of all middle-aged and older women in the United States and many European countries regularly use vitamin D supplements,^3^^,^^4^ believing that additional vitamin D reduces their future fracture risk and improves their health. Serum 25-hydroxyvitamin D concentration (S-25OHD, an integrated marker of vitamin D exposure) has also been related to the rate of falls,^5^^,^^6^ an important determinant of fragility fractures.^7^ Nonetheless, randomized controlled trials examining vitamin D supplementation for preventing falls, bone loss, and fractures in community-dwelling individuals have generally shown no clinically significant effects.^6^, ^7^, ^8^, ^9^, ^10^ There is a strong need to understand who needs higher body stores of vitamin D and vitamin D supplementation. It is essential to establish an accurate definition of vitamin D deficiency and improve our knowledge of the long-term health consequences of seasonal fluctuation in vitamin D levels, which aligns with the previous recommendations of leading research institutes.^11^

Previous randomized controlled trials (RCT)s examining the effect of vitamin D supplements on BMD have lasted up to five years; however, compliance is a concern in long-term RCTs.^8^^,^^9^^,^^11^ Therefore, long-term longitudinal observational studies with repeat measurements of both BMD and S-25OHD are needed to better understand the impact of vitamin D status on BMD loss and how longitudinal changes in S-25OHD influence age-related BMD loss. Only one such study has been published, mainly including vitamin D-replete (mean S-25OHD ≈80 nmol/L) Australian women and men examined six years apart.^12^ No clear association between changes in S-25OHD and bone loss was found, and it is still unknown if there is an association among individuals with low vitamin D levels.

Circulating vitamin D metabolite concentrations are highly dependent on the season. The primary source of vitamin D is synthesis in the skin from ultraviolet B radiation from sunlight. The strength of radiation depends on season and latitude,^13^ but at latitudes >40°–covering most of Europe–ultraviolet B radiation for vitamin synthesis is insufficient during dark seasons.^14^ Mean decreases in S-25OHD concentrations from sunny season peak to nadir in late winter can reach 50%,^15^^,^^16^ a seasonality seen both at high latitudes and in more temperate climates, i.e., Southern Europe and Australia.^15^^,^^16^ However, it is not established whether low S-25OHD during the year’s dark and sunny seasons has a similar clinical interpretation.^13^ Our previous analysis of cross-sectional S-25OHD data suggests that summer concentrations of S-25OHD may be more informative than winter concentrations to predict BMD^13^ and future fracture risk.^17^ We therefore hypothesize that in longitudinal data, long-term constant low vitamin D status in the sunny season will have a greater impact on bone health changes over time than long-term constant low vitamin D status in the dark season. Further, we hypothesize that increasing S-25OHD over time will improve BMD to a greater extent if low S-25OHD levels are initially measured in the sunny season compared to the dark season.

Therefore, we used a recently finalized longitudinal population-based cohort of women with an average of 12 years of follow-up to examine longitudinally stable categories of S-25OHD alongside follow-up total hip areal BMD (aBMD). We also investigated whether changes in S-25OHD during follow-up influence changes in aBMD based on different baseline S-25OHD levels and the season. An overview of the study is presented in the graphical abstract.

## Patients and Methods

We used a previously described subcohort^13^^,^^17^ of women from the population-based Swedish Mammography Cohort, which is part of the Swedish Infrastructure for Medical Population-Based Life-Course and Environmental Research (SIMPLER; https://www.simpler4health.se/). From 1987-1990, all women born 1914-1948 living in Uppsala County (n=48,517) and all women born between 1917-1948 living in Västmanland County (n=41,786) were asked to complete a comprehensive food frequency questionnaire when invited to mammography screening. Completed questionnaires were obtained from 66 651 (74%) individuals. From November 3, 2003 to September 3, 2009, we invited a random subcohort of Swedish Mammography Cohort participants living in Uppsala city or the surrounding area, to participate in a clinical examination. From February 5, 2015 to May 22, 2019, this subcohort, including women born 1920-1948 and living in the county of Uppsala (latitude 58^o^N), was invited to participate in a second clinical examination. In total, 1809 women (60% of those eligible) participated in the second examination. At both clinical examinations the women underwent dual-energy X-ray absorptiometry (DXA) measurements, fasting blood and urine sample collection, fat biopsies, and anthropometric assessment. The biological samples were collected all year long, which means that the season of sample collection could be different for each individual at the first and second examination. At each examination, the blood sample was collected in the morning following an overnight fast; the samples were then protected from light and spun in a refrigerated centrifuge, frozen in multiple tubes, and stored at −80 °C until analysis. One month before each clinical examination, participants responded to lifestyle, food frequency, and health questionnaires, which they had also completed in 1987-1990, 1997, 2008-2009, and 2019. The study has ethical approvals from ethical review boards; each participant provided written informed consent.

### Laboratory Measurements

Using identical methods, we successfully examined 1802 samples from the first examination cycle of the subcohort in one batch in 2013 and 1802 samples from the second examination cycle in 2023. The 2 primary forms of vitamin D are vitamin D_2_ and vitamin D_3_. The more abundant form, vitamin D_3_ (cholecalciferol), is synthesized in the skin from 7-dehydrocholesterol and is also derived from foods (naturally occurring and in fortified products) and supplements. Both forms of vitamin D are considered biologically inactive until they undergo two enzymatic hydroxylation reactions. The first occurs in the liver and is mediated by 25-hydroxylase, which forms 25-dihydroxyvitamin D (25OHD), also named calcidiol. This is the major circulating vitamin D metabolite and the primary determinant of vitamin D status.^18^ Serum 25OHD is a useful integrated marker of vitamin D exposure, incorporating endogenous synthesis from solar exposure, dietary intake from foods, fortified products, and supplements. The second hydroxylation in the synthesis mainly occurs in the kidney and is mediated by 1α-hydroxylase, which converts 25OHD to the biologically active hormone 1,25-dihydroxyvitamin D (1,25[OH]_2_D), or calcitriol. The renal synthesis of 1,25(OH)_2_D is tightly regulated by 2 counteracting hormones, with up-regulation of synthesis by parathyroid hormone (PTH) and down-regulation by fibroblast-like growth factor-23 (FGF23).

Total S-25OHD, including S-25OHD_3_ and S-25OHD_2_, was determined in our study with high-performance liquid chromatography (HPLC) with atmospheric pressure chemical ionization and mass spectrometry (MS) at Vitas, Oslo, Norway (www.vitas.no), as previously described,^13^ with minor differences in the methods for the analysis of baseline and follow-up samples. Specifically, 50 μL of human plasma was diluted with 100 μL isopropanol with deuterium-labeled 25-OH-vitamin D3 as the internal standard. After thorough mixing (15 min) and centrifugation (10 min, 4000 g at 10 °C), an aliquot of 20 μL was injected from the supernatant into the HPLC system. The HPLC was performed with an Agilent 1260/1290 liquid chromatograph (Agilent Technologies) interfaced by atmospheric pressure chemical ionization to an Agilent Technologies 6465B Triple Quad LC-MS/MS operated in multiple reaction monitoring mode. Vitamin D analogs were separated on a Phenomenex Kinetex PFP 150 mm × 4.6 mm column with 2.6 μM particles. The column temperature was 40 °C. A one-point calibration curve was made from analysis of natural plasma calibrator, wherein the value was set using National Institute of Standards and Technology-certified reference material (SRM-1950). The method is linear from at least 5 to 400 nM, with 95% recovery, and the detection limit is 1-4 nM. The coefficients of variation for inter-assay analyses were between 5% and 8%. The Vitamin D External Quality Assessment Scheme (DEQAS; https://www.deqas.org/) accredits the method, which is highly accurate (95%) compared with target values obtained from the DEQAS scheme. The technique has also been standardized against serum provided by the US National Institute of Standards and Technology.^19^ We report S-25OHD values in nmol/L. To convert from nmol/L to ng/mL, divide the nmol/L value by 2.496.

We determined S-1,25-(OH)_2_D using IDS-iSYS 1,25-VitD-Xp (Immunodiagnostic Systems).^13^ To determine free S-25OHD_3_, we used the Free 25OH Vitamin D ELISA (DIAsource) per the manufacturer’s instructions. Plasma parathyroid hormone, FGF23, calcium, phosphate, creatinine, cystatin C, albumin, C-reactive protein, alanine aminotransferase, β CrossLaps, and osteocalcin were analyzed using routine methods as previously described.^13^ We estimated the absolute estimated glomerular filtration rate based on age, weight, height, plasma creatinine, and cystatin C, as previously described.^13^

### Body Composition

All women in the study underwent body composition measurements with DXA scans. The mean total hip areal BMD (aBMD; bilateral scans), as well as total fat and lean body mass, were measured using the same DXA equipment (Lunar Prodigy; GE Medical Systems), and all measurements from 2003 until the end of follow-up were done by the same DXA-accredited research nurse.^13^ On the basis of duplicate measurements with repositioning according to recommendations from the International Society for Clinical Densitometry, the short-term precision measurement error varied between 0.8% and 1.5%, depending on the measurement site. The long-term coefficient of variation from 2003 through 2023 was <1% for a spine phantom. In connection with the examination, body weight and height were measured. Body mass index was calculated as weight (in kilograms)/height (in meters).^2^

### Assessment of Cohort Characteristics and Potential Confounders

We collected lifestyle information from questionnaires, including smoking habits, sedentary behavior, moderate leisure time physical activity and exercise, educational level, living alone, postmenopausal estrogen therapy, use of corticosteroids, bone-specific medication, vitamin D and calcium supplement use, multivitamin and mineral supplement use, menopausal status, and parity (see details in supplementary materials). Nutrient intakes were estimated as cumulative averages from 3 food frequency questionnaires (in 1987, 1997, 2009, and 2019), and one month before the research reception visits/time of blood draw. Diagnosis codes were collated from the National Patient Register to calculate a weighted Charlson comorbidity index.

The Swedish National Prescribed Drug Register contains data on all prescriptions dispensed to the Swedish population from July 2005 onward. Using anatomical therapeutic chemical codes, we collected information on all dispensation dates through 2022 for different types of bisphosphonates, denosumab, vitamin or calcium supplements, estrogen replacement therapy, and corticosteroid use.

After DNA extraction from whole blood samples and genotyping using Illumina’s Global Screening Array (v1), we constructed a polygenetic risk score for S-25OHD using the seven most influential single nucleotide polymorphism determinants of S-25OHD variance.^20^ We used this to help describe our study sample.

### Statistical Analyses

#### Monthly Mean and Cumulative Distribution in S-25OHD at Baseline and Follow-up

We descriptively show the mean total S-25OHD for all individuals with blood samples collected in the same month and at the same examination cycle to describe seasonal patterns. This was then plotted against the month of the blood draw. We also plotted the cumulative distribution of S-25OHD at baseline and follow-up and the change in S-25OHD from baseline to follow-up.

#### Confirmation of Previous Cross-Sectional Results

We previously showed that low S-25OHD during the sunny season, but not the dark season, is strongly linked cross-sectionally to low aBMD.^13^ We set out to confirm that these associations persist when the sample is restricted to individuals who contributed longitudinal data. Using baseline data, we estimated the marginal means of hip aBMD by the following 5 categories of S-25OHD (<40, 40-50, 50-60, 60-70, and >70 nmol/L) from multivariable-adjusted linear regression models, using S-25OHD >70 nmol/L as the reference. Analyses were stratified into 2 categories by season of blood draw,^17^ where May through October was considered the sunny season and November through April was considered the dark season. The categorization was based on the peak and nadir of S-25OHD levels, which were in August and February/March, respectively.^17^

#### The Season-Average aBMD at Baseline and Follow-up, and Relative Change in aBMD by Baseline S-25OHD Categories

Crude and multivariable-adjusted absolute baseline and follow-up total aBMD and relative mean changes in hip aBMD over 12 years of follow-up were estimated against the five S-25OHD categories. These results are presented without season stratification.

#### Relative Difference in aBMD in Women Belonging to the Same Category of S-25OHD During the 12-Year Follow-up

In the first main analysis of this study, we evaluated the impact of long-term constant levels of S-25OHD on aBMD after restricting the longitudinal data to women belonging to the same S-25OHD category at both baseline and follow-up. We calculated multivariable-adjusted relative differences in marginal mean hip aBMD at the end of follow-up for each S-25OHD category with >70 nmol/L as the reference category, stratified by baseline dark or sunny season.

#### Long-Term Differences in S-25OHD and Long-Term Change in aBMD, Overall and by Season of Blood Draw

In the second main analysis of this study, we further estimated the relative change (%) in total hip aBMD during the 12-year follow-up per 20 nmol/L change in S-25OHD (corresponding to ∼1 SD difference in S-25OHD) during the same period, both overall and by the 5 categories of baseline S-25OHD (<40, 40-50, 50-60, 60-70, and >70 nmol/L). We used age-adjusted and multivariable-adjusted linear regression models, and the multivariable model was additionally adjusted for baseline hip aBMD.

Subsequently, we restricted the analysis to women who had increased their S-25OHD during follow-up (49% of the total sample) to mimic an interventional effort to improve vitamin D status. We estimated the relative multivariable-adjusted linear regression change (%) in total hip aBMD during the 12-year follow-up per 20 nmol/L increase in S-25OHD. Analyses were stratified by 4 different cut-offs for low baseline vitamin D status (<45, <50, <55, and <60 nmol/L) and by the baseline dark or sunny season of blood draw, with the multivariable model additionally adjusted for baseline total hip aBMD. We could not use <40 nmol/L S-25OHD as a cut-off since few women in our study with that level at baseline had a substantial season-stratified increase in S-25OHD during follow-up. On the basis of results from randomized controlled trials and as a comparison of our chosen exposure unit of 20 nmol/L change in S-25OHD, an oral supplemental dose of 400 IU (10 μg)/day of vitamin D led to an average 32 nmol/L increase in S-25OHD from a mean baseline concentration of 33 nmol/L (6 studies).^8^ The change with a dose of 800 IU (20 μg)/day averaged 35 nmol/L higher S-25OHD, from a mean of 44 nmol/L (7 studies).^8^ Accordingly, an increase of S-25OHD by 20 nmol/L corresponds to the effect of a moderately large vitamin D supplementation dose.

#### Long-Term Change in S-25OHD and Long-Term Change in Circulating Biomarkers

Finally, we used multivariable-adjusted linear regression models to estimate the relative change (%) in circulating PTH, bone turnover markers, calcium, and phosphate during the 12-year follow-up per 20 nmol/L change in S-25OHD during the same period. This multivariable model included baseline biomarker values. We present both the overall results and results stratified by the baseline S-25OHD categories.

The multivariable models included baseline and follow-up values for age (continuous), 4 seasons of blood draw (summer, fall, winter, and spring), body mass index (continuous), height (continuous), any bisphosphonate use, alendronate use, use of denosumab, bisphosphonate use within 2 years before the second examination, denosumab use within 2 years before the second examination, energy intake (continuous), calcium intake (continuous), reading/watching TV (continuous and 6 response levels), physical exercise (continuous and 5 response levels), walking/biking (continuous and 6 response levels), absolute estimated glomerular filtration rate (continuous), and weighted Charlson comorbidity index (continuous). Additional adjustments for baseline educational level (3 categories), living alone (yes vs no), smoking status (never, former, and current), nulliparity, use of calcium supplementation, and menopausal age (continuous) did not substantially affect our estimates. We used SAS 9.4 (SAS Institute, Cary) and Stata version 15.1 (StataCorp, College Station) for the statistical calculations. The study has ethical approvals from the ethical review boards in Uppsala and Stockholm, Sweden (approval numbers: 110/92, 99/070, 03-484, 2017/222, 2020-04172. Informed consent was sought for all participants.

## Results

Of the 1802 women included in the study, the mean age at baseline was 65 years. The mean time from the baseline to the follow-up clinical examinations (ie, measurements of S-25OHD and BMD) was 12.2 years (SD 1.3) with a range of 8.7 to 18.6 years. On both occasions, approximately one-tenth of women had an S-25OHD value below 40 nmol/L, and 25% had a concentration higher than 70 nmol/L (Table). About 5% of women had S-25OHD concentrations <40 nmol/L during the sunny season at baseline, with a corresponding figure of 8% at follow-up. These proportions were 16% and 13% in the dark season. We observed higher S-1,25(OH)_2_D_3_, vitamin D binding protein, and FGF23 values with higher S-25OHD, whereas plasma parathyroid hormone was lower. None of the women at baseline or follow-up with S-25OHD below 50 nmol/L had possible biochemical osteomalacia, defined as the presence of low albumin-adjusted S-Ca (<2.0 mmol/L) and elevated PTH (>6.8 pmol/L).^21^ Lifestyle factors, such as alcohol and energy intake, smoking status, and physical activity, were evenly distributed by categories of S-25OHD, and this was also evident for the polygenetic vitamin D risk score.

**Table:** Baseline and Follow-up Characteristics of the Women by S-25OHD Categories at Baseline^a^^,^^b^

Characteristics of participants	Categories of S-25OHD (nmol/L) at baseline				
	<40	40-50	50-60	60-70	>70
No. of women (%)	201 (11)	322 (18)	405 (22)	385 (21)	489 (27)
N examined in the dark season (%)	155 (16)	213 (22)	220 (23)	196 (20)	175 (18)
N examined in the sunny season (%)	46 (5)	109 (13)	185 (22)	189 (22)	314 (37)
Continuous variables					
Age (y)	65.0 (4.8)	64.6 (4.7)	65.0 (5.2)	64.4 (4.3)	65.1 (4.9)
S-25OHD (nmol/L)	33.3 (5.9)	45.2 (2.9)	54.9 (2.8)	64.8 (3.0)	82.0 (10.1)
S-25OHD_3_ (nmol/L)	32.9 (6.0)	44.6 (3.6)	54.3 (3.3)	64.4 (3.2)	81.4 (10.6)
S-25OHD_2_ (nmol/L)	0.4 (1.4)	0.6 (2.0)	0.6 (1.7)	0.4 (1.4)	0.6 (2.8)
S-1,25(OH)_2_D3 (pg/mL)	39.2 (19.2)	41.0 (18.7)	41.3 (15.9)	NA	56.0 (22.7)
Free S-25OHD_3_ (pg/mL)	4.34 (1.71)	5.13 (2.23)	5.84 (2.32)	6.15 (2.34)	7.0 (2.20)
S-FGF23 (pg/mL)	37.9 (15.3)	43.4 (15.4)	45.7 (12.0)	44.6 (15.5)	43.7 (17.3)
Body mass index (kg/m^2^)	26.6 (4.5)	26.0 (4.0)	25.7 (3.9)	25.0 (3.6)	24.1 (3.4)
Height (cm)	164.4 (6.4)	164.7 (5.9)	164.2 (5.5)	165.0 (5.6)	165.0 (3.4)
Weight (kg)	71.7 (12.1)	70.6 (10.9)	69.3 (11.4)	68.1 (10.4)	65.9 (9.8)
Lean mass (kg)	40.2 (4.5)	40.1 (4.2)	39.8 (4.1)	39.8 (3.9)	39.4 (4.1)
Fat mass (kg)	28.2 (8.8)	27.6 (8.3)	26.5 (8.5)	25.3 (8.0)	23.6 (7.5)
Fat percent (%)	38.6 (7.1)	38.3 (6.5)	37.4 (6.6)	36.4 (6.9)	35.0 (6.8)
P-PTH (pmol/L)	5.63 (1.91)	5.20 (1.60)	4.95 (1.56)	4.73 (1.46)	4.51 (1.37)
P-calcium (mmol/L)	2.28 (0.09)	2.30 (0.11)	2.30 (0.11)	2.31 (0.11)	2.31 (0.10)
P-phosphate (mmol/L)	1.15 (0.14)	1.16 (0.13)	1.15 (0.13)	1.17 (0.14)	1.17 (0.13)
P-CRP (mg/L)	2.7 (5.2)	2.1 (2.2)	2.5 (5.4)	2.8 (7.7)	2.2 (3.9)
S-albumin (mmol/L)	0.63 (0.04)	0.62 (0.09)	0.66 (0.07)	0.65 (0.05)	0.64 (0.04)
P-Alanine transaminase (μkat/L)	0.23 (0.13)	0.24 (0.14)	0.23 (0.14)	0.24 (0.14)	0.23 (0.12)
eGFR (mL/min/1.73m^2^)	88.4 (13.0)	89.0 (12.8)	85.9 (13.9)	86.2 (13.4)	84.4 (12.7)
Charlson comorbidity index (score)	0.07 (0.28)	0.10 (0.38)	0.14 (0.55)	0.10 (0.41)	0.11 (0.43)
Menopausal age (y)	49.9 (3.8)	49.8 (3.4)	49.9 (3.10)	50.0 (3.2)	50.0 (3.0)
Dietary vitamin D intake (μg/d)	4.4 (1.0)	4.6 (1.0)	4.7 (1.0)	4.6 (1.0)	4.7 (1.0)
Total vitamin D intake (μg/d)	5.1 (2.0)	5.7 (2.7)	5.8 (2.5)	5.9 (2.5)	6.3 (2.8)
Dietary calcium intake (mg/d)	1022 (217)	1011 (204)	992 (211)	1010 (183)	1022 (202)
Alcohol intake (g/wk)	5.7 (5.4)	5.5 (4.3)	5.8 (4.3)	6.1 (5.0)	6.8 (5.3)
Energy intake (kcal/d)	1702.00	1717 (364)	1742 (372)	1789 (371)	1749 (366)
H/wk spent outdoors	8.7 (6.6)	14.1 (40.7)	13.1 (10.3)	15.5 (13.0)	15.3 (12.7)
H/d reading/watching TV	2.6 (0.7)	2.6 (0.8)	2.5 (0.7)	2.5 (0.6)	2.5 (0.6)
H/wk exercising	2.8 (1.2)	2.8 (1.2)	2.8 (1.2)	2.9 (1.2)	3.0 (1.1)
Min/d walking/cycling	39 (27)	37 (25)	41 (27)	42 (27)	44 (29)
Polygenetic vitamin D risk score effect (nmol/L)	5.06 (1.56)	4,81 (1.56)	4.82 (1.49)	5.01 (1.53)	4.90 (1.52)
Category variables, number (%)					
Never smoker	96 (48)	172 (53)	224 (56)	218 (57)	259 (53)
Former smoker	90 (45)	130 (40)	152 (38)	141 (37)	205 (42)
Current smoker	15 (7)	20 (6)	26 (6)	26 (7)	25 (5)
Vitamin D supplement use	15 (7)	27 (8)	54 (13)	72 (19)	109 (22)
Calcium supplement use	9 (5)	23 (7)	48 (12)	53 (14)	101 (21)
Multivitamin/mineral supplement use	10 (5)	16 (5)	39 (10)	56 (15)	66 (13)
Bone-specific medication baseline	0	3 (1)	8 (2)	3 (1)	26 (5)
Ever estrogen replacement therapy	135 (67)	217 (67)	262 (65)	260 (67)	340 (69)
Ever use of corticosteroids	14 (7)	27 (8)	31 (8)	30 (8)	46 (9)
Living alone	69 (34)	89 (28)	122 (30)	117 (30)	144 (29)
Nulliparity	33 (16)	43 (13)	37 (9)	43 (11)	53 (11)
Low educational level	63 (31)	138 (43)	167 (42)	148 (39)	209 (43)
Medium educational level	21 (10)	30 (9)	37 (9)	36 (9)	40 (8)
High educational level	117 (58)	154 (48)	198 (49)	201 (52)	240 (49)

	Categories of S-25OHD (nmol/L) at Follow-up				
	<40	40-50	50-60	60-70	>70
No. of women (%)	198 (11)	323 (18)	431 (24)	394 (22)	456 (25)
N examined in the dark season (%)	137 (13)	205 (20)	255 (24)	217 (21)	228 (22)
N examined in the sunny season (%)	61 (8)	118 (16)	176 (23)	177 (23)	228 (30)
Continuous variables					
Age (y)	77.4 (4.8)	77.2 (4.7)	77.1 (4.7)	76.8 (4.7)	76.9 (4.8)
Perceived age by nurse (y)	75.3 (5.7)	74.8 (5.8)	74.0 (5.8)	73.2 (6.0)	73.2 (6.8)
S-25OHD (nmol/L)	33.7 (5.5)	45.8 (2.7)	55.2 (2.9)	64.9 (2.9)	83.3 (13.3)
S-25OHD_3_ (nmol/L)	32.8 (6.0)	44.9 (3.9)	54.1 (4.1)	64.2 (3.8)	82.5 (13.7)
S-25OHD_2_ (nmol/L)	0.9 (2.6)	0.9 (2.8)	1.1 (2.9)	0.7 (2.4)	0.8 (2.1)
S-1,25(OH)_2_D3 (pg/mL)	32.0 (9.8)	33.0 (10.0)	34.4 (10.9)	34.7 (10.6)	37.7 (12.0)
Body mass index (kg/m^2^)	26.7 (4.6)	26.5 (4.3)	25.9 (4.2)	25.3 (4.2)	24.5 (3.9)
Height (cm)	163.1 (6.5)	162.4 (6.2)	163.3 (5.6)	163.5 (5.9)	162.9 (6.1)
Weight (kg)	70.9 (13.0)	69.9 (11.5)	68.2 (11.8)	67.7 (11.8)	65.0 (10.9)
Lean mass (kg)	38.8 (4.5)	38.7 (3.9)	38.6 (3.8)	38.7 (4.0)	38.0 (4.1)
Fat mass (kg)	28.5 (9.9)	27.6 (8.8)	26.1 (9.2)	25.3 (9.3)	23.5 (8.7)
Fat percent (%)	39.1 (7.8)	38.6 (7.0)	37.3 (7.4)	36.4 (8.0)	35.1 (8.2)
P-PTH (pmol/L)	5.19 (1.95)	4.70 (1.60)	4.54 (1.73)	4.35 (1.82)	4.14 (1.64)
P-calcium (mmol/L)	2.36 (0.09)	2.35 (0.10)	2.36 (0.15)	2.36 (0.13)	2.37 (0.11)
P-phosphate (mmol/L)	1.11 (0.13)	1.12 (0.13)	1.11 (0.12)	1.13 (0.13)	1.11 (0.12)
P-CRP (mg/L)	2.1 (2.1)	2.4 (4.0)	2.3 (3.3)	2.5 (6.4)	2.4 (4.6)
P-albumin (g/L)	38.1 (2.8)	37.6 (2.6)	37.7 (3.0)	37.9 (2.8)	37.9 (2.7)
P-Alanine transaminase (μkat/L)	0.33 (0.50)	0.31 (0.17)	0.29 (0.13)	0.31 (0.24)	0.29 (0.22)
eGFR (mL/min/1.73m^2^)	76.4 (15.9)	75.7 (17.6)	74.9 (18.9)	73.0 (16.2)	71.6 (16.7)
Charlson comorbidity index (score)	0.51 (1.08)	0.37 (0.97)	0.33 (0.74)	0.41 (0.96)	0.44 (0.97)
Dietary vitamin D intake (μg/d)	6.7 (2.0)	6.9 (2.2)	6.9 (2.1)	7.1 (2.2)	7.0 (2.5)
Total vitamin D intake (μg/d)	7.0 (3.5)	7.7 (4.0)	7.9 (4.1)	8.6 (4.6)	10.2 (6.8)
Dietary calcium intake (mg/d)	1112 (287)	1091 (288)	1137 (280)	1134 (294)	1163 (312)
Energy intake (kcal/d)	1772 (492)	1916 (518)	1867 (515)	1870 (529)	1867 (512)
H/wk spent outdoors, summer	5.1 (1.1)	5.2 (1.1)	5.3 (1.0)	5.2 (1.1)	5.3 (1.2)
H/wk spent outdoors, winter	3.8 (1.6)	4.0 (1.5)	4.0 (1.7)	4.0 (1.5)	3.8 (1.7)
H/d reading/watching TV	3.2 (0.9)	3.0 (0.7)	2.9 (0.7)	2.9 (0.7)	2.9 (0.7)
H/wk exercising	2.6 (1.3)	2.7 (1.3)	2.7 (1.3)	2.7 (1.3)	2.7 (1.4)
Min/d walking/cycling	40 (27)	43 (27)	48 (30)	50 (31)	49 (30)
Polygenetic vitamin D risk score effect (nmol/L)	2.53 (1.33)	2.35 (1.31)	2.26 (1.16)	1.88 (1.21)	1.63 (1.05)
Category variables, number (%)					
Current smoker	8 (4)	20 (6)	23 (5)	18 (2)	24 (5)
Abstainer alcohol	13 (7)	28 (9)	30 (7)	24 (6)	30 (7)
Vitamin D supplement use	0 (0)	2 (1)	4 (1)	8 (2)	24 (4)
Calcium and vitamin D supplements	34 (17)	54 (17)	110 (26)	114 (29)	206 (45)
Multivitamin/mineral supplements	35 (18)	89 (29)	144 (35)	156 (41)	232 (53)
Bone-specific medication after baseline	9 (5)	13 (4)	49 (4)	49 (12)	90 (20)
Ever use of alendronate	11 (6)	13 (4)	52 (12)	51 (13)	93 (20)
Ever use of other bisphosphonates	1 (1)	0 (0)	2 (1)	2 (1)	4 (1)
Ever use of denosumab	1 (1)	2 (1)	16 (4)	18 (5)	29 (2)
Current use of estrogen replacement	2 (1)	4 (1)	5 (1)	4 (1)	6 (1)
Current use of corticosteroids	1 (1)	6 (2)	8 (2)	5 (2)	6 (1)
Use sunscreen when sunny					
Always	8 (4)	10 (3)	24 (6)	34 (9)	32 (7)
Most of the time	78 (39)	101 (32)	106 (25)	81 (21)	98 (22)
Sometimes	112 (57)	206 (65)	298 (70)	276 (71)	319 (71)
Never	0 (0)	0 (0)	0 (0)	0 (0)	2 (1)

a Abbreviations: S-25OHD, serum 25-hydroxyvitamin D.

b Data are mean ± SD or n (%).

### Monthly Mean and Cumulative Distribution in S-25OHD at Baseline and Follow-up

**Figure 1:**
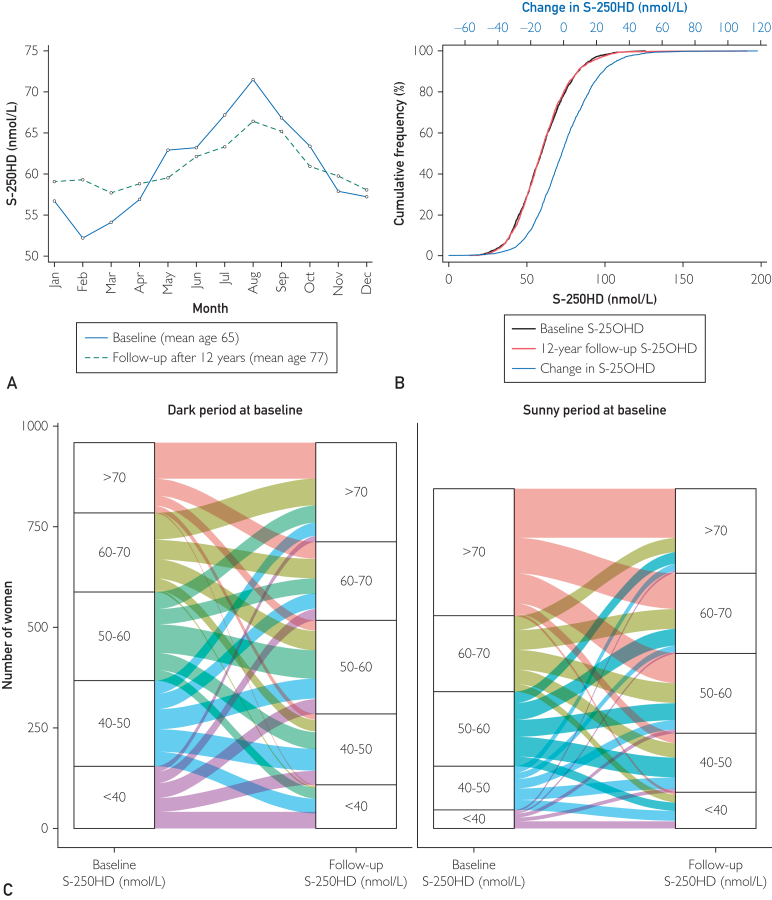
Serum 25-hydroxyvitamin D (S-25OHD) by month at baseline and follow-up (A) and cumulative frequency of change in S-25OHD from baseline to follow-up (B). In (C), we display changes in S-25OHD categories during follow-up by baseline season of blood draw.

We found a peak concentration of S-25OHD in August at both examinations and a nadir in late winter (February-March, Figure 1A). Interestingly, the seasonal fluctuation in S-25OHD was more apparent at baseline than at follow-up. It should be noted that twice as many women used vitamin D supplementation at the second examination (Table). The cohort’s overall S-25OHD distributions were similar at baseline and follow-up, although the women were 12 years older (Figure 1B). The mean concentration was 60.2 (SD 17.3) nmol/L at baseline and 60.4 (SD 17.7) nmol/L at the follow-up. About 13% had lowered their S-25OHD by more than 20 nmol/L, whereas 14% had increased their concentration by more than 20 nmol/L (Figure 1B). In Figure 1C, we display changes in S-25OHD categories during follow-up by baseline season of blood draw.

### Confirmation of Previous Cross-Sectional Results

We confirmed our previous cross-sectional finding with a 0.05 g/cm^2^ (5%) lower baseline aBMD at the total hip in those with sunny season S-25OHD levels <40 nmol/L versus those with >70 nmol/L (*P*=.007) (Supplemental Figure 1, available online at http://www.mcpiqojournal.org). Such a difference was not observed with samples collected during the dark season (*P*=.62).

### The Season-Average aBMD at Baseline and Follow-up, and Relative Change in aBMD by Baseline S-25OHD Categories

**Figure 2:**
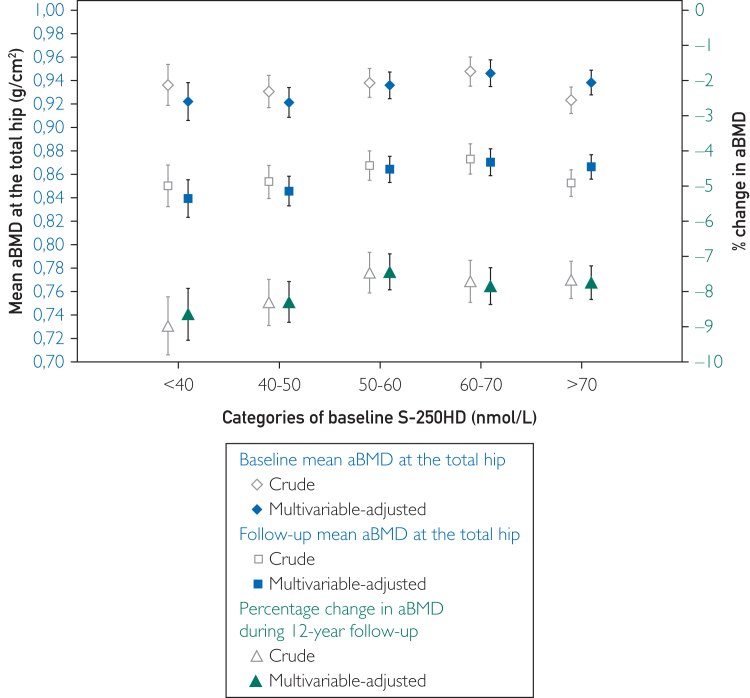
Crude and multivariable-adjusted mean total hip areal BMD (aBMD) by serum 25-hydroxyvitamin D (S-25OHD) categories, at baseline and follow-up, and crude and multivariable-adjusted change in total hip aBMD during follow-up. The multivariable model was adjusted for baseline and follow-up age, season (December-February, March-May, June-August, and September-November), body mass index, height, any bisphosphonate use, alendronate use, use of denosumab, bisphosphonate use within 2 years before the second examination, denosumab use within 2 years before the second examination, energy intake, calcium intake, reading/watching TV (6 response levels), leisure time physical exercise (5 response levels), walking/biking (continuous and 6 response levels), eGFR, and weighted Charlson comorbidity index. The number of women in each category, from low to high S-25OHD in the dark season was 156 (<40 nmol/L), 213, 219, 196, 175, and in the sunny season 46 (<40 nmol/L), 109, 185, 189, and 314. Error bars represent 95% confidence intervals. eGFR, estimated glomerular filtration rate

During the 12-year follow-up, the women, on average, lost 7.9% (95% CI, 7.6-8.2) of their aBMD at the total hip (Figure 2). Without stratification on the season, we observed only modest differences in average loss of total hip aBMD depending on baseline vitamin D status. Those with baseline S-25OHD <40 nmol/L lost a multivariable-adjusted 8.6% (95% CI, 7.9-9.4), and those with S-25OHD >70 nmol/L lost 7.7% (95% CI, 7.3-8.2) of their hip aBMD (*P*-value .048 for the modest 0.9% difference).

### Relative Difference in aBMD in Women Belonging to the Same Category of S-25OHD During the 12-Year Follow-up

**Figure 3:**
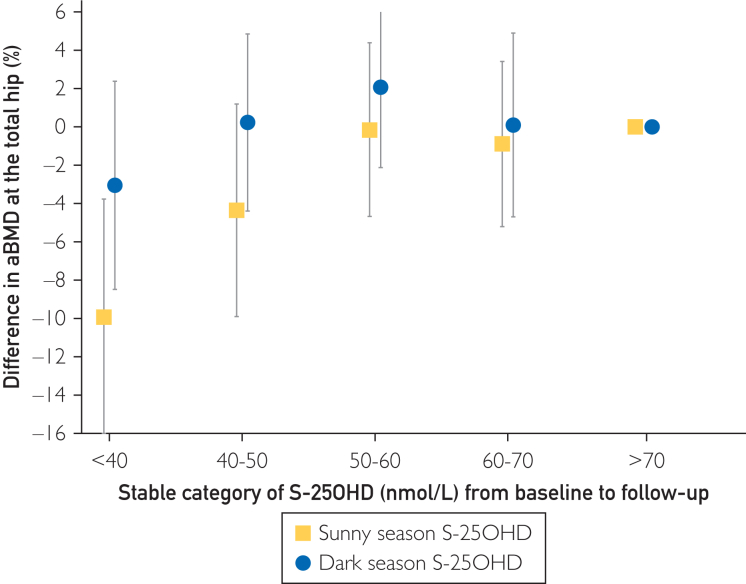
Multivariable-adjusted difference in total hip areal BMD (aBMD) at follow-up in percent, for women in the same category of serum 25-hydroxyvitamin D at baseline and 12-year follow-up (ie, stable category of S-25OHD) compared with the highest category of stable S-25OHD (>70 nMol/L). The results are presented by baseline S-25OHD category and season of blood draw. The multivariable model was adjusted for baseline and follow-up age, season (December-February, March-May, June-August, and September-November), body mass index, height, any bisphosphonate use, alendronate use, use of denosumab, bisphosphonate use within 2 years before the second examination, denosumab use within 2 years before the second examination, energy intake, calcium intake, reading/watching TV (6 response levels), leisure time physical exercise (5 response levels), walking/biking (continuous and 6 response levels), eGFR, and weighted Charlson comorbidity index. The number of women in each category, from low to high S-25OHD in the dark season was 41 (<40 nmol/L), 56, 71, 48, 90, and in the sunny season, 19 (<40 nmol/L), 28, 41, 49, and 121. Error bars represent 95% confidence intervals.

Using our longitudinal data, we examined differences in total hip aBMD at follow-up in women (n=562) who belonged to the same S-25OHD category at baseline and follow-up by the season of baseline blood draw (Figure 3). For women with baseline blood draws in the sunny season, those with constant S-25OHD <40 nmol/L displayed 0.086 g/cm^2^ lower total hip aBMD values (95% CI, 0.033-0.139; *P*=.002) than those with constant S-25OHD >70 nmol/L, corresponding to a 10.0% difference (95% CI, 3.8%-16.1%). This was threefold larger than a corresponding comparison for individuals with baseline samples drawn during the dark season (Figure 3)

### Long-Term Differences in S-25OHD and Long-Term Change in aBMD, Overall and by Season of Blood Draw

**Figure 4:**
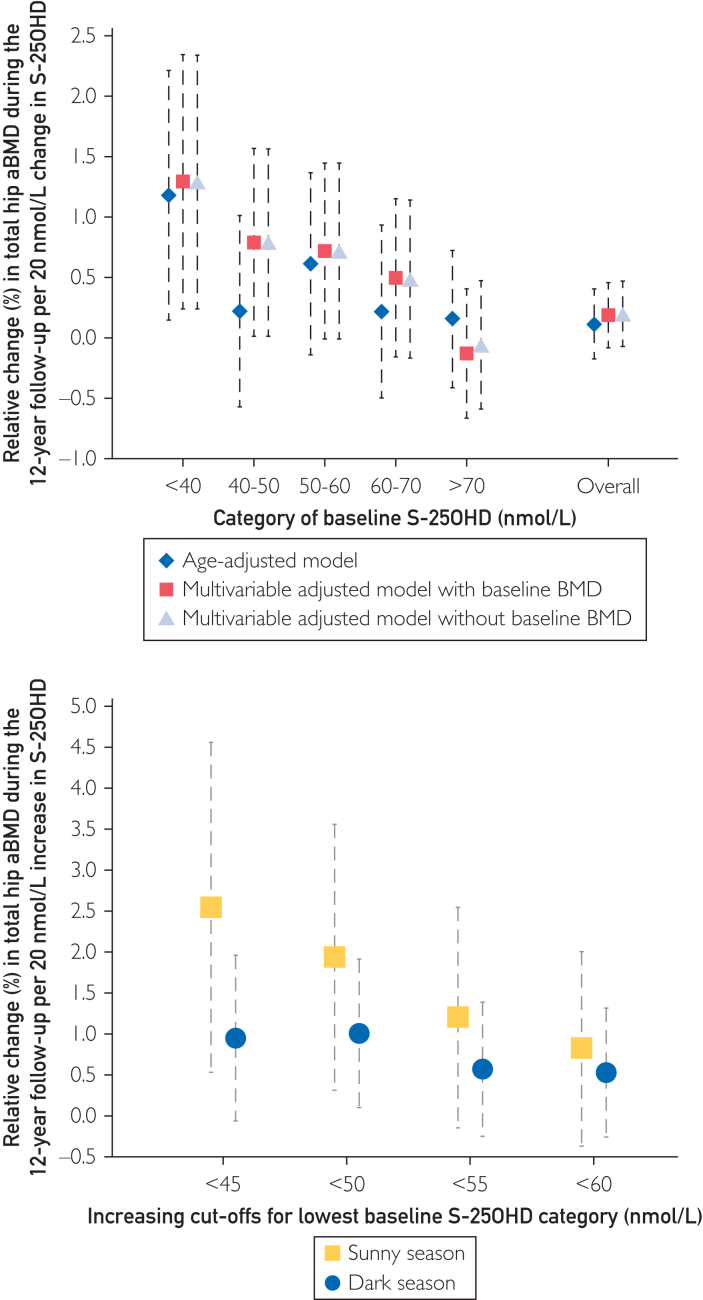
The crude and multivariable-adjusted 12-year percentage change in total hip areal BMD (aBMD) after any 20 nmol/L change (reduction or increase) in serum 25-hydroxyvitamin D during follow-up (Panel A) by baseline vitamin D status. Panel B displays the impact of a 20 nmol/L increase in S-25OHD on the multivariable-adjusted percentage change in total hip aBMD by season (sunny season May-October; dark season November-April) and baseline S-25OHD categories. The primary multivariable model was adjusted for baseline and follow-up age, season (December-February, March-May, June-August, and September-November), body mass index, height, any bisphosphonate use, alendronate use, use of denosumab, bisphosphonate use within 2 years before the second examination, denosumab use within 2 years before the second examination, energy intake, calcium intake, reading/watching TV (6 response levels), leisure time physical exercise (5 response levels), walking/biking (continuous and 6 response levels), eGFR, and weighted Charlson comorbidity index. A second multivariable-adjusted model additionally included total hip aBMD at baseline. The number of women with S-25OHD below 45 nmol was n=361, below 50 nmol/L n=524, below 55 nmol/L n=745, and below 60 nmol/L n=928. The number of women who increased S-25OHD during follow-up with a baseline <45 nmol/L was 237 in the dark and 91 in the sunny season. The corresponding numbers with a baseline S-25OHD <50 nmol/L were 322 and 130; baseline S-25OHD <55 nmol/L 408 and 197; and baseline S-25OHD <60 nmol/L 470 and 250. Error bars represent 95% confidence intervals. eGFR, estimate glomerular filtration rate.

Next, we estimated the impact of a 20 nmol/L change in S-25OHD during the 12-year follow-up period on changes in hip aBMD, overall, and stratified by baseline S-25OHD categories (Figure 4A). On average, a change in S-25OHD did not confer a meaningful change in hip aBMD (0.2%; 95% CI, −0.1% to 0.5%). However, women with S-25OHD below 40 nmol/L at baseline displayed a moderate increase in multivariable-adjusted hip aBMD by 1.3% (95% CI, 0.2-2.4; *P*=.02) per 20 nmol/L change in S-25OHD. The result remained essentially similar after adjustment for baseline aBMD. This estimate was successively attenuated with increasing categories of baseline S-25OHD.

We refined this analysis by examining the impact of a unidirectional 20 nmol/L *increase* in S-25OHD on multivariable-adjusted changes in hip aBMD by 4 increasing cut-offs for low baseline S-25OHD, from <45 nmol/L to <60 nmol/L. The results tended to differ according to the baseline season of the blood draw (Figure 4B). Women with sunny season S-25OHD below 45 nmol/L at baseline displayed a positive change in hip aBMD during follow-up of 2.5% (95% CI 0.5-4.6; *P*=.01) per 20 nmol/L increase in S-25OHD. The corresponding increase in hip aBMD with a cut-off of <50 nmol/L was 1.9% (95% CI, 0.3-3.6; *P*=.02) per 20 nmol/L higher S-25OHD. This estimate was further attenuated with cut-offs at <55 nmol/L and at <60 nmol/L for the low S-25OHD category. These described increases in aBMD in women following increases in S-25OHD in women with low S-25OHD during the sunny season should be seen in the perspective that the average woman in the cohort lost 8% of aBMD during the 12-year follow-up period, as shown in Figure 2.

### Long-Term Change in S-25OHD and Long-Term Change in Circulating Biomarkers

We also evaluated the change in serum PTH, bone turnover markers, calcium, and phosphate with an increase during follow-up in S-25OHD. On average, PTH decreased by 4% per 20 nmol/L higher S-25OHD (*P*<.0001); a reduction was observed irrespective of baseline vitamin D status (Supplemental Figure 2, available online at http://www.mcpiqojournal.org). In parallel, calcium slightly increased by 0.4% (*P*=.0037) per 20 nmol/L higher S-25OHD, and a raise was observed irrespective of baseline vitamin D status. In contrast, osteocalcin, CrossLaps, and phosphate remained unaffected after increased S-25OHD.

## Discussion

This is the first longitudinal cohort study to examine the association between long-term changes in S-25OHD and long-term changes in hip aBMD. We show that women with S-25OHD concentrations below 40 nmol/L for 12 years during the sunny season have approximately 10% lower hip aBMD than women with high levels. A 20 nmol/L increase in S-25OHD, corresponding to a moderate dose of vitamin D supplementation, during the same period in women with baseline sunny season S-25OHD <45 nmol/L confers a clinically meaningful improved hip aBMD. The estimated 2.5% (0.4 SD) improvement in hip aBMD per 20 nmol/L increase in S-25OHD corresponds to a theoretical 20% reduction in hip fracture risk.^8^^,^^22^ The clinical relevance of this result is further demonstrated when we consider that bisphosphonate treatment vs placebo leads to 3% femoral neck BMD gain, a halved vertebral fracture risk, and a 20%-30% reduction in nonvertebral fracture risk.^22^ Only about 5% of the women in our cohort had S-25OHD <40 nmol/L in the summer, as we previously showed using the entire baseline cohort.^17^ This subgroup seems to be suitable for interventional efforts.

With a growing number of older individuals, the number of fragility fracture cases is expected to double over the next 40 years, presenting significant challenges to healthcare and the well-being of older populations. Preventing fragility fractures is essential, and targeting at-risk individuals with adequate interventions is key. One-third to half of Western adult populations use vitamin D regularly, believing it reduces their future fracture risk and generally improves their health.^23^ Despite the uncertainty of defining deficiency, more than 10 million blood vitamin D concentration tests are performed annually in the United States.^23^ The annual global market for vitamin D supplements and blood vitamin D tests is valued at USD 2.3 billion. However, surprisingly, vitamin D supplementation has not shown the expected effects on osteoporosis and prevention of fragility fractures,^8^ and high doses of vitamin D supplements may even reduce bone mass.^24^ Moreover, previous Mendelian randomization analyses have not found a causal relationship between S-25OHD and BMD^25^ or fracture risk,^26^ but this design is not optimal for detecting threshold effects. Secondary analyses of 2 RCTs^27^^,^^28^ indicate 0.9% and 0.8% improvements in total hip BMD with vitamin D supplementation (monthly doses of vitamin D_3_ 100,000 IU [2500 μg] during 2 years^27^^,^^28^ or daily doses of 1000 IU/day [25 μg/day] during one year)^27^^,^^28^ in individuals with considerably lowered vitamin D status (<30 nmol/L) during the year’s dark season. This modest effect is not expected given the massive vitamin D supplement treatment effect of the nowadays unusual diagnoses of rickets and osteomalacia.^11^^,^^18^

We have previously shown that dark season concentrations of S-25OHD are unrelated to aBMD or future fracture risk.^13^^,^^17^ In contrast, summer measurements show a clear dose-response relationship where low S-25OHD levels are linked to lower aBMD, risk of osteoporosis, and future higher fracture rates.^13^^,^^17^ Importantly, those without a sufficient augmentation of S-25OHD in the summer will be left with low concentrations throughout the year, causing a health impact that is mainly avoided in those with a shorter period of deficient S-25OHD that occurs only during late winter. Our findings indicate that only 1 out of 20 individuals in the older community-dwelling population is at particular risk of low BMD because of low S-25OHD (<40 nmol/L) in the sunny season. The clinical practice, however, focuses on measuring S-25OHD during the year's dark season, not the sunny season. No RCT has used low summer concentrations of S-25OHD as the inclusion criterion.

With our country’s northern location (northernmost point 69^o^N, southernmost point 55^o^N), many fear that we are especially vulnerable to a lack of vitamin D. Still, we seem, on average, to have higher serum levels of S-25OHD compared with Southern Europe, also in samples taken during the winter.^13^^,^^17^ Theoretically, such results can be explained by food fortification with vitamin D in Scandinavian settings and more intense tanning behavior during the sunny season, leading to high body stores of vitamin D or a genetic predisposition to effective endogenous vitamin D synthesis.

Of interest, a reduction in circulating PTH was observed with higher S-25OHD during follow-up, irrespective of baseline vitamin D status. Lowered serum PTH concentrations have also regularly been found in vitamin D supplementation trials, even though no effect on BMD has been demonstrated. Meta-analysis of RCTs has also displayed that PTH decreases linearly during vitamin D supplementation at any given S-25OHD level,^29^ indicating that a reduction in PTH after vitamin D supplementation is a blunt instrument to predict an effect on bone health. Raised serum calcium from normal levels after increases in S-25OHD in our study, both at low and high baseline S-25OHD, can be a concern given that a genetically determined modest raise in serum calcium confers a higher risk of myocardial infarction.^30^

Strengths of our study include the long-term longitudinal design and richness of the cohort, the population-based design, the same DXA equipment and research nurse during 20 years of examination, and the use of S-25OHD measured with a high-accuracy gold-standard method.^13^ There was no indication that women with low S-25OHD concentrations, compared to those with high concentrations, had different genetics, lifestyle habits, or were frail individuals with derangements in biomarkers other than those related to the vitamin D axis.^17^ Related to that, women with low S-25OHD were more frequent sunscreen users, less often spent time in the sun, and less frequent vitamin D supplement users. Moreover, changes in body weight and body composition during follow-up were largely similar across categories of changes in S-25OHD.

Our study also has limitations. In some circumstances, body composition can affect the DXA aBMD results, since body size can impact the distance between the DXA bed and the measured bone. In addition, increased body fat may cause BMD to be overestimated in obese individuals. However, we used a Lunar Prodigy narrow fan beam DXA, which corrects the scans to the actual effective object plane, thus limiting the impact of body composition on the DXA results. Magnification errors that occur with ordinary fan beam DXA are smaller when using a narrow fan beam along the axis in the Lunar Prodigy. Furthermore, all measurements were made by the same DXA-accredited x-ray nurse, using the same DXA equipment. These steps help to limit the impact of measurement error on our results. Our analyses were adjusted for several conceivable covariates without substantially impacting our estimates. Still, residual confounding remains a limitation. For example, domains of physical activity not captured by our questionnaire, as well as socioeconomic factors that could impact both vitamin D levels and BMD via unanticipated pathways that are not accounted for in the adjustment set, may contribute to residual confounding. Moreover, individual repeated S-25OHD measurements throughout one year would have been valuable. Few had extremely low S-25OHD, and an even larger longitudinal cohort would have been preferential. A larger study size could also have enabled a stratified analysis by 4 seasons, not only a dichotomization of sunny and dark seasons. Moreover, women with low vitamin D status during the summer will probably also have, on average, lower S-25OHD concentrations during the dark season. Still, the predictive ability of a low dark season value will be masked by the 3-4 times higher proportion of women whose levels are only temporarily low during winter.^13^ Accordingly, a dark season measurement will not be informative regarding vitamin D status over the whole year, and the exposure contrast between sunny and dark season measurements of vitamin D will be diluted by the mix of individuals with temporally low vitamin D values in the dark season and those with low values throughout the whole year. We studied only Caucasian, fair-skinned women in Sweden, and our findings may not apply to other ethnicities. Finally, the women in our cohort were mostly calcium-replete, and our findings may not be relevant in settings with lower calcium intake.

Our findings have implications for evaluating vitamin D status in clinical practice and shed light on the lack of effect seen with vitamin D supplementation in many BMD trials. We conclude that sunny season concentrations of S-25OHD are essential when targeting interventional efforts with vitamin D supplementation to improve bone health.

## Potential Competing Interests

The authors report no competing interests
