# Supplementary material for: Long-Term Change in Vitamin D Status and its Association With Change in Total Hip Bone Mineral Density in Older Women: A Population-Based Cohort Study
Source: Mayo Clin Proc Innov Qual Outcomes. 2025 Dec 3;10(1):100681. doi: 10.1016/j.mayocpiqo.2025.100681 (PMC12720181; doi:10.1016/j.mayocpiqo.2025.100681)
Supplement: Supplemental Material [file mmc1.pdf]

Supplementary materials

Long-term change in vitamin D status and its association with change in total hip bone mineral density in older women: a population-based cohort study

Karl Michaëlsson,[1] Håkan Melhus,[2] Liisa Byberg,[1] Eva Warensjö Lemming,[1] Bodil Svennblad,[1] Jonas Höijer,[1] Hannah L Brooke[1]

[1] Medical Epidemiology, Department of Surgical Sciences, Uppsala University, SE-751 35 Uppsala, Sweden, Karl Michaëlsson (0000-0003-2815-1217) professor, Liisa Byberg (0000-0002-4421-6466) professor, Eva Warensjö Lemming (0000-0002-0149-452x) associate professor, Bodil Svennblad (0000-0001-6393-3439) statistician, Jonas Höijer (0000-0002-0012-1211) statistician, Hannah Brooke (0000-0002-7085-1551) associate professor,

[2] Clinical Pharmacology, Department of Medical Sciences, Uppsala University, S-751 35 Uppsala Sweden, Håkan Melhus (0000-0002-6857-5973) professor

Correspondence to:

Dr Hannah L Brooke

Department of Surgical Sciences

Uppsala University

751 35 Uppsala

Sweden

E-mail: Hannah.brooke@uu.se

Questions used for descriptive characteristics and potential confounding variables

Smoking Habits

Have you ever smoked cigarettes regularly (more than 5 cigarettes/week):

1=No

2=Yes, currently

3=Yes, but I stopped

Categorised as Never smoker, Former smoker, Current smoker

Physical Activity and Exercise

Your physical activity in **the past month**

Walking and cycling

Almost never

< 20 min/day

20-40 min

40-60 min

1-1.5 hours

>1.5 hours/day

Exercise (e.g. going to the gym)

Almost never

<1 hour/week

1 hour/week

2-3 hours/week

4-5 hours/week

>5 hours/week

Reading or watching TV

<1 hour/day

1-2 hours/day

3-4 hours/day

5-6 hours/day

7-8 hours/day

6=>8 hours/day

These variables were included as continuous variables in multi-variable adjusted models

Educational Level

What level of education do you have?

Primary school (≤9 years)

High school (10-12 years)

College/University (≥12 years)

Living alone Status

There are _____ people in the household.

I have lived alone for ___ years

Based on these questions we created a binary variable to indicate if individuals live alone (Yes/No)

Medication use

Postmenopausal Estrogen Therapy as systemic estradiol treatment with ATC code G03CA03 collected at the participants examination

Use Of Corticosteroids

Cortisone in tablet form or inhalation

Never

Sometimes

Regularly

Self-reported or ATC code H02AB

Bone-Specific Medication

Bisphosphonate use was identified from the Swedish National Prescribed Drug Register using ATC codes starting with M05BA or M05BB, and for denosumab use, we used ATC

code M05BX04. The dates of the prescriptions were used to further categorize bisphosphonate and denosumab use, to use before baseline, use during follow-up, and use only within the two last years of follow-up. Bisphosphonate use was further separated into alendronate use or other bisphosphonate use.

Dietary Supplement Use

Vitamin D

Never

Sometimes

Regularly

Self-reported or ATC-codes A11CC or A12AX

Calcium

Never

Sometimes

Regularly

Self-reported or ATC codes A12A

Multivitamins with or without minerals

Never

Sometimes

Regularly

Self-reported or ATC codes A11A or A11B

We created a binary variable to indicate if individuals used each of these dietary supplements (Yes/No)

Menopausal Status

At what age did you enter menopause?

Continuous variable

Parity

Do you have any children? (Yes/No)

Supplementary Figure 1

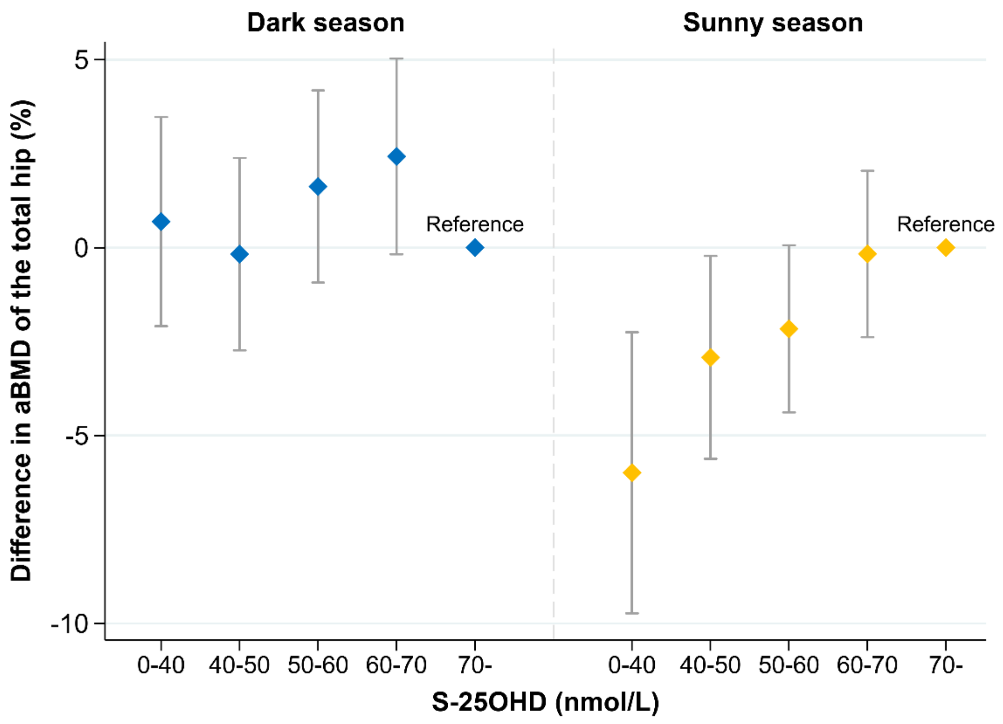

Supplementary Figure 1. Multivariable adjusted association between baseline serum 25-hydroxyvitamin D (S-25OHD) and total hip areal bone mineral density (aBMD), by season of blood draw. The results are presented as differences compared with the highest category of S-25OHD (>70 nMol/L). The multivariable model included baseline age, season of blood draw (December-February, March-May, June-August, September-November), body mass index, height, any bisphosphonate use, alendronate use, use of denosumab, energy intake, calcium intake, reading/watching TV (six response levels), leisure time physical exercise (five response levels), walking/biking (six response levels), eGFR, and weighted Charlson comorbidity index. The number of women in each category, from low to high S-25OHD in the dark season was 156 (<40 nmol/L), 213, 219, 196, 175, and in the sunny season 46 (<40 nmol/L), 109, 185, 189, and 314. Error bars represent 95% confidence intervals.

Supplementary Figure 2

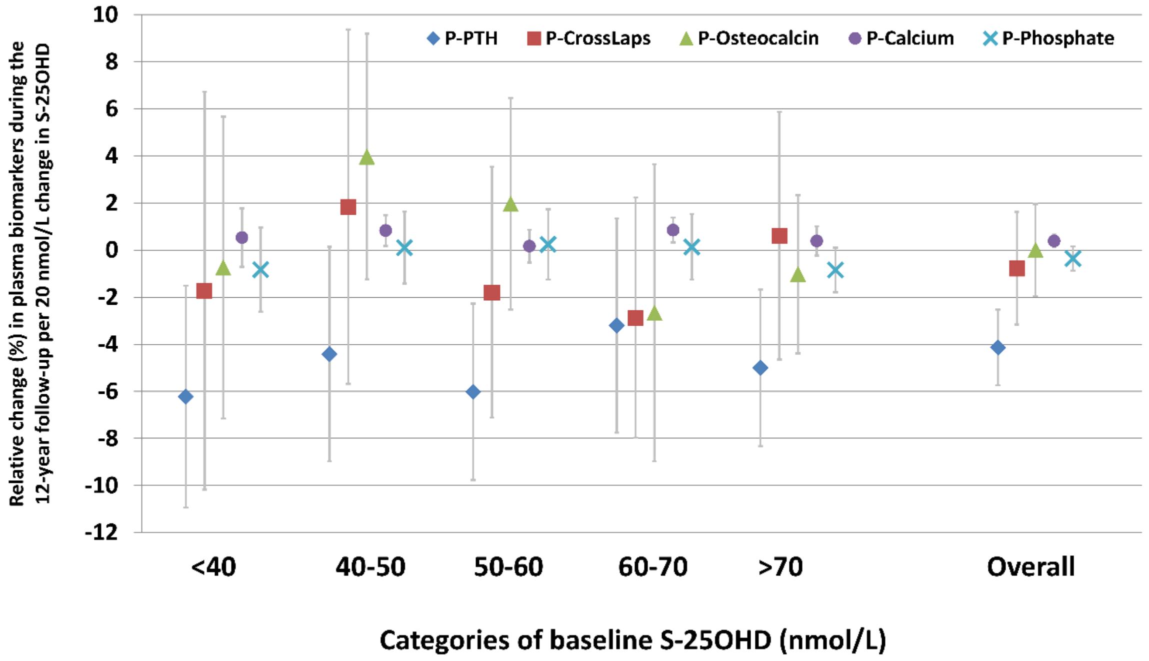

Supplementary Figure 2. Multivariable-adjusted change in serum PTH, bone turnover markers, calcium, and phosphate with a 20 nmol/L increase during follow-up in S-25OHD, overall, and by baseline S-25OHD categories. The multivariable model was adjusted for baseline and follow-up age, season (December-February, March-May, June-August, September-November), body mass index, height, any bisphosphonate use, alendronate use, use of denosumab, bisphosphonate use within two years before the second examination, denosumab use within two years before the second examination, energy intake, calcium intake, reading/watching TV (six response levels), leisure time physical exercise (five response levels), walking/biking (continuous, six response levels), eGFR, and weighted Charlson comorbidity index. The number of women in each category, from low to high S-25OHD in the dark season was 156 (<40 nmol/L), 213, 219, 196, 175, and in the sunny season 46 (<40 nmol/L), 109, 185, 189, and 314. Error bars represent 95% confidence intervals.
